# Hepatitis B Vaccination History among medical intern doctors and nurses in three national referral hospitals in Uganda

**DOI:** 10.4314/ahs.v25i4.8

**Published:** 2025-12

**Authors:** Anna Maria Gwokyalya, Innocent Nakityo, Nelson Twinamasiko, Raymond Bernard Kihumuro, Isaac Isiko, Ronald Mutebi Kasoma, Felix Bongomin

**Affiliations:** 1 Department of Medicine, School of Medicine, College of Health Sciences, Makerere University, Kampala, Uganda; 2 Makerere Lung Institute, Department of Medicine, School of Medicine, Makerere University, Kampala, Uganda; 3 Mbarara University of Science and Technology; 4 Department of Community Medicine, Institute of Medical Sciences and Research, Nims University, Jaipur, India; 5 Department of Medicine, Mengo Hospital, Uganda; 6 Department of Medical Microbiology and Immunology, Faculty of Medicine, Gulu University, Gulu, Uganda

**Keywords:** Hepatitis B Virus (HBV), HBV Vaccination, medical intern

## Abstract

**Background:**

Healthcare trainees are at high risk of acquiring the Hepatitis B Virus (HBV).

**Objective:**

We aimed to evaluate the HBV vaccination status among medical intern doctors and nurses in Uganda.

**Methods:**

We conducted a cross-sectional study of medical interns (doctors and nurses) working in three national referral hospitals in Uganda. Self-reported vaccination status was classified into “fully vaccinated”, “partially vaccinated,” and “not vaccinated.”

**Results:**

A total of 186 participants were enrolled in the study. More than half of the participants were from Mulago National Referral Hospital (n=110, 59.14%), were male (n=103, 55.38%), and were medical intern doctors (n=127, 68.28%). The majority of the medical interns (n=166, 89.0%) had received at least one dose, but n=97 (58.43%) reported receiving all three recommended doses of the HBV vaccine. Forgetfulness (n=8, 30.8%) and cost of the vaccine (n=8, 30.8 %) were the main reasons some participants were unvaccinated.

**Conclusion:**

The completion rate of the HBV vaccination schedule among medical interns was low. It is essential to establish systems that require medical interns to have completed their HBV vaccination before starting their medical internship.

## Introduction

Hepatitis B is a viral infection that affects the liver and can lead to both acute and chronic infection. According to the World Health Organization (WHO), an estimated 254 million people were living with chronic hepatitis B infection in 2022, with 1.2 million new infections occurring each year^1^. Africa accounts for 25% of the global hepatitis B burden, and Uganda is among the countries with a high prevalence, reported at 4.3% (5.6 % among men and 3.1% among women) as of 20 1 8^2^. The Hepatitis B Virus (HBV) can be transmitted in two main ways: vertically, from mother to child, or horizontally, through exposure to the blood and bodily fluids (semen or vaginal fluids) of infected individuals. Transmission can occur through needle stick injuries, accidental splashes of body fluids, or sexual intercourse with multiple partners^3^,^4^. Healthcare workers (HCW), particularly trainees, face an increased risk of contracting the hepatitis B virus (HBV) due to frequent occupational exposure to blood and bodily fluids^5^,^6^.

All graduate doctors, dentists, nurses, and pharmacists in Uganda are expected to complete a one-year internship at a designated hospital before obtaining a practicing license^7^. Medical interns play a crucial role in the health workforce, providing essential first-line healthcare services in many hospitals. However, inten doctors and nurses often face challenging working conditions, including inadequate resources, poor safety standards, and a lack of supervision or support from consultants^8^. These factors increase their risk of occupational exposure, particularly during surgical procedures.

In 2002, Uganda's Ministry of Health (MOH) incorporated early childhood vaccination against hepatitis B into the Uganda National Expanded Program on Immunization (UNEPI)^9^. Despite this initiative, healthcare workers who were born before this policy was implemented missed out on vaccination. Consequently, the MOH recommended that all healthcare professionals, including medical interns, receive the hepatitis B virus (HBV) vaccine preferably before commencing their clinical duties to safeguard against potential exposure. The standard MOH vaccination protocol administers the HBV vaccine in three doses at 0, 1, and 6 months^9^. However, there is a lack of follow-up studies specifically evaluating the HBV vaccination coverage among medical interns in Uganda—a group particularly at risk of exposure to HBV during their training in various clinical settings. This study aimed to assess the self-reported HBV vaccination histories of medical interns, including both doctors and nurses, at three national referral hospitals in Uganda. Assessing their vaccination status is crucial for enhancing infection prevention strategies and ensuring the safety of healthcare providers during their formative years in the medical profession.^10^

## Methods

### Study design

We conducted a descriptive cross-sectional study from 2nd October to 13th December 2022, employing quantitative data collection methods.

### Study Setting and Study Population

The study was conducted in three national referral hospitals in Kampala district that receive the greatest number of medical interns in Uganda, that is, Mulago National Referral Hospital (MNRH), Kawempe National Referral Hospital, and Kiruddu National Referral Hospital. A total of 319 medical interns (164 doctors and 155 nurses) were deployed in 2021/2022; 199 in MNRH, 98 in Kiruddu NRH, and 114 in Kawempe NRH.

### Sample size and sampling

The estimated sample size was calculated using the StatCal option of Epi Info version 7. Considering a confidence level of 95% and a margin of error of 5%, with the prevalence of fully vaccinated healthcare workers (HCWs) at 57.8%^10^, the estimated sample size was 176 participants. We conducted three visits to each facility and recruited participants based on their availability and willingness to participate in the study. During each visit, we found that intern doctors were more readily available, as they typically worked 8 to 12-hour shifts from Monday to Friday, while intern nurses had shorter and less frequent shifts.

### Data collection procedures

Participants completed a pretested self-administered questionnaire consisting of two parts: i) demographic characteristics of respondents and ii) self-reported HBV vaccination history. The questionnaire underwent pre-testing with ten medical interns at Kawempe National Referral Hospital, and their responses were not included in the final data analysis. The irregularities identified during this process were reviewed and addressed by a physician from the Department of Medicine at Makerere University before approving the refined and finalized version of the questionnaire.

### Statistical analysis

The outcome variable is the self-reported vaccination status of the medical interns. Individuals were described as either “not vaccinated” (received 0 doses), “partially vaccinated” (received 1 or 2 doses), or “fully vaccinated” (received three doses). Completed questionnaires were exported into Microsoft Excel 2016 for cleaning. The cleaned data were then transferred to R version 4.3.1. Numerical data were summarized as means, standard deviations, and medians. Categorical data were summarized as frequencies and proportions. First, bivariate analyses were performed to assess the association between outcome variables and independent variables. The independent variables were age, sex, profession, the institution of training, location of the institution, and internship site. Univariable and multivariable logistic regression were performed to adjust for confounders and estimate the independent predictors of the vaccination status of medical interns. Variables in the multivariable regression were selected based on the association of the particular variable with the outcome variable, as well as previous data from other studies. All tests were two-tailed, and a P-value < 0.05 was considered significant.

## Results

### Demographic characteristics

Of the 186 medical interns, n=103 (55.38%) were males and n=82 (44.09%) were more than 27 years old, with a mean age of 27.4 (±4.19). The majority of the participants were medical intern doctors, n=127 (68.28%) and n=110 (59.14%) were training at MNRH (Table 1).

**Table 1:** Demographic characteristics of medical interns

Characteristic	Frequency	Percentage (%)
**Age**		
<25	34	18.28
25-27	70	37.63
>27	82	44.09
**Sex**		
Male	103	55.38
Female	83	44.62
**Profession**		
Intern Doctor	127	68.28
Intern Nurse	59	31.72
**Training Institution**		
Makerere University	61	32.8
Mbarara University	35	18.82
Kampala International University	30	16.13
Busitema University	10	5.38
Gulu University	7	3.76
Others	43	23.12
**Location of training institution**		
Central	82	44.09
Western	74	39.78
Northern	16	8.6
Eastern	12	6.45
Outside Uganda	2	1.08
**Internship site**		
Mulago National Referral Hospital	110	59.14
Kiruddu National Referral Hospital	54	29.03
Kawempe National Referral Hospital	22	11.83
**Nationality**		
Ugandan	181	97.31
Non-Ugandan	5	2.69

### HBV Vaccination history of medical interns

More than three-quarters of the participants, n=166 (89.00%), reported having received one dose of the HBV vaccine, but n=97 (58.43%) reported having received all three recommended doses. Forgetfulness (n=8, 30.80%) and cost of the vaccine (n=8, 30.80%) were the main reasons some participants were not vaccinated (Table 2).

**Table 2:** Responses of participants to questions on the vaccination status of medical interns

Question	Response	Frequency N (%)
**Number of doses required (N=186)**	Three	159 (85.48%)
	Two	26 (14.0%)
	One	1 (0.54%)
**Received the HBV vaccine (N=186)**	Yes	166 (89.24%)
	No	20 (10.75%)
	One	15 (9.04%)
**Number of doses received (N=166)**	Two	54 (32.53%)
	Three	97 (58.43%)
**Reason for receiving the vaccine (N=166)**	Peer pressure	4 (2.41%)
	Fear of HBV Infection	115 (69.28%)
	I know its complications	1 (0.60%)
	Requirement by the Ministry of Health and the university	42 (25.30%)
	Required to be vaccinated before clinical rotations	1 (0.60%)
	Other	3 (1.81%)
**Reason for not receiving the vaccine (N=26)**	Forgetfulness	8 (30.8%)
	No interest	3 (11.5%)
	The vaccine is expensive	8 (30.8%)
	Others	7 (26.9%)

### Factors associated with the vaccination status of medical interns

After bivariate and multivariable regression analysis, there were no factors associated with the vaccination status of medical interns (Table 3).

**Table 3:** Regression analysis of factors associated with the vaccination status of medical interns

Variable		Fully vaccinated	Not vaccinated	Partially vaccinated	OR (univariable)	OR (multivariable)
Sex	Female	67 (80.7)	3 (3.6)	13 (15.7)	-	-
	Male	86 (83.5)	3 (2.9)	14 (13.6)	0.83 (0.39-1.77, p=0.623)	-
Age	<25	29 (85.3)	2 (5.9)	3 (8.8)	-	-
	25-27	57 (81.4)	0 (0.0)	13 (18.6)	1.32 (0.45-4.45, p=0.626)	-
	>27	67 (81.7)	4 (4.9)	11 (13.4)	1.30 (0.45-4.29, p=0.642)	-
Marital status	Married	29 (78.4)	1 (2.7)	7 (18.9)	-	-
	Single	124 (83.2)	5 (3.4)	20 (13.4)	0.73 (0.31-1.88, p=0.491)	-
Profession	Intern doctor	106 (83.5)	2 (1.6)	19 (15.0)	-	-
	Intern Nurse	47 (79.7)	4 (6.8)	8 (13.6)	1.29 (0.57-2.80, p=0.528)	-
Nationality	Non-Ugandan	3 (60.0)	1 (20.0)	1 (20.0)	-	-
	Ugandan	150 (82.9)	5 (2.8)	26 (14.4)	0.31 (0.05-2.43, p=0.210)	0.32 (0.05-2.56, p=0.229)

Number in dataframe = 186, Number in model = 186, Missing = 0, AIC = 176.6, C-statistic = 0.602, H&L = Chi-sq (8) 0.00 (p=1.000)

## Discussion

It is essential to emphasize that despite the availability of a safe and effective HBV vaccine worldwide, many healthcare workers in developing countries remain at risk because a significant portion of them are not fully vaccinated against HBV infection^11^. The percentage of fully vaccinated HCWs in a systematic review and meta-analysis of HBV studies in African countries ranges between 13.4 to 62.1%^12^. In our study, we discovered that most medical interns received the first dose of the vaccine, but only 58.43% completed the recommended three doses. In a similar study done at Gulu Regional Referral Hospital based in Northern Uganda, more than half (57.9%) of the healthcare workers were vaccinated against HBV infection, but 42.9% had received the three doses of the vaccine, both values turning out lower than what we observed in our study^13^. Additionally, the completion rate in our study was just slightly higher than that found in another study done among HCWs in the Wakiso district, where 57.8% of the HCWs had completed the HBV vaccination schedule^10^. Previous studies among HCWs in Kenya, Tanzania, and Nigeria revealed even lower completion rates of 48%^14^, 33.6%^15^, and 48.9%^16^. The unsatisfactory completion of the HBV vaccination schedule is attributed to the high cost of the vaccine, vaccine unavailability, busy work schedules, underestimation of the risk of infection, lack of time, fear of side effects, forgetfulness, and the extended vaccination schedule^15^–^18^. In our study, forgetfulness and vaccine cost contributed to the low HBV vaccination completion rate. The government of Uganda has mitigated these challenges by incorporating the HBV vaccine in the childhood vaccination schedule since 2002, thus improving the vaccine coverage among healthcare workers born after this intervention^9^. In our study, more than 80% of medical interns were born before 2002. It is therefore imperative to increase awareness about the importance of the HBV vaccination and improve the availability of the vaccine to all unvaccinated healthcare workers.

Previous studies state that the three-dose series of Hepatitis B vaccines confer full protection in more than 90% of healthy recipients aged < 40 years, and a considerable proportion of the HCWs who received one or two doses are still at risk of contracting HBV infection in case of exposure^19^. Therefore, increasing publicity and awareness about the importance of completing the vaccination is pertinent to improving vaccination coverage among medical interns. In a study done at two hospitals in Pakistan, healthcare workers stated job entry requirements as the primary reason behind the successful completion of the vaccination schedule^20^. It is important to establish systems that require all medical interns, especially those born before 2002, to receive the three HBV vaccine doses before commencing their training, and for all medical interns, regardless of birthdate to provide credible proof of HBV vaccination before their deployment into various health facilities.

The medical profession, age, and sex have been noted as predictors for the HBV vaccine uptake among healthcare workers and trainees^10^,^16^,^18^,^21^. However, these associations were not observed in our study.

### Strengths and Limitations

We selected medical interns who spared time off their duties to participate in the study, which could have introduced a selection bias, making findings not entirely representative of all medical interns in those facilities. Moreover, our findings may not be generalized to all medical interns nationwide since we studied only three health facilities. Moreover, in this study, information regarding the screening of medical interns before receiving the HBV vaccine was not obtained, and HBV serological markers to determine immunity from natural exposure or from the vaccine were not tested. Nonetheless, this is the first study to assess self-reported HBV vaccination status among medical interns.

## Conclusion and Recommendations

While most medical interns reported having received at least one dose of the HBV vaccine, the completion rate of all three doses was low. Given the occupational exposure risk to HBV infection, it is essential to ensure that all medical interns have proof of HBV vaccination before the commencement of their training. This can be a requirement at the Uganda Medical and Dental Practitioners Council (UMDPC) or the individual health facilities.
